# Expression of the COVID‐19 receptor ACE2 in the human conjunctiva

**DOI:** 10.1002/jmv.25981

**Published:** 2020-07-11

**Authors:** Clemens Lange, Julian Wolf, Claudia Auw‐Haedrich, Anja Schlecht, Stefaniya Boneva, Thabo Lapp, Ralf Horres, Hansjürgen Agostini, Gottfried Martin, Thomas Reinhard, Günther Schlunck

**Affiliations:** ^1^ Eye Center, Medical Center, Faculty of Medicine University of Freiburg Freiburg Germany; ^2^ GenXPro Frankfurt Innovation Center Biotechnology Frankfurt Germany

**Keywords:** *ACE2*, COVID‐19, human conjunctiva, SARS‐CoV‐2, *TMPRSS2*

## Abstract

SARS‐CoV‐2 is assumed to use angiotensin‐converting enzyme 2 (*ACE2*) and other auxiliary proteins for cell entry. Recent studies have described conjunctival congestion in 0.8% of patients with laboratory‐confirmed severe acute respiratory syndrome coronavirus‐2 (SARS‐CoV‐2), and there has been speculation that SARS‐CoV‐2 can be transmitted through the conjunctiva. However, it is currently unclear whether conjunctival epithelial cells express ACE2 and its cofactors. In this study, a total of 38 conjunctival samples from 38 patients, including 12 healthy conjunctivas, 12 melanomas, seven squamous cell carcinomas, and seven papilloma samples, were analyzed using high‐throughput RNA sequencing to assess messenger RNA (mRNA) expression of the SARS‐CoV‐2 receptor *ACE2* and its cofactors including *TMPRSS2, ANPEP, DPP4*, and *ENPEP*. ACE2 protein expression was assessed in eight healthy conjunctival samples using immunohistochemistry. Our results show that the SARS‐CoV‐2 receptor *ACE2* is not substantially expressed in conjunctival samples on the mRNA (median: 0.0 transcripts per million [TPM], min: 0.0 TPM, max: 1.7 TPM) and protein levels. Similar results were obtained for the transcription of other auxiliary molecules. In conclusion, this study finds no evidence for a significant expression of *ACE2* and its auxiliary mediators for cell entry in conjunctival samples, making conjunctival infection with SARS‐CoV‐2 via these mediators unlikely.

## 1 INTRODUCTION

The outbreak of coronavirus disease (COVID‐19) caused by severe acute respiratory syndrome coronavirus‐2 (SARS‐CoV‐2) from December 2019 in Wuhan, Hubei, China, has been declared a global public health emergency. Patients infected with SARS‐CoV‐2 develop symptoms of fever, cough, and fatigue that can quickly progress to pneumonia. SARS‐CoV‐2 is highly infectious and transmitted mainly by inhaling droplets or aerosols released by an infected individual and possibly by a feco‐oral transmission route.^1^ Recent studies have described “conjunctival congestion” in 9 of 1099 patients (0.8%) with laboratory‐confirmed SARS‐CoV‐2^2^ and some clinicians have speculated that the disease can be transmitted through the conjunctiva^3^, ^4^, ^5^ highlighting the need for further investigations on transmission pathways.

Similar to other coronaviruses, SARS‐CoV‐2 uses angiotensin‐converting enzyme 2 (ACE2) protein to gain entry into cells.^6^, ^7^ Since the outbreak, many studies described *ACE2* expression across human tissues, including lung, stomach, ileum, colon, liver, and kidney,^8^, ^9^ supporting the clinical observation that SARS‐CoV‐2 can infect multiple organs. Interestingly, Zou et al^9^ reported that alveolar type 2 cells (the proposed main target cell of SARS‐CoV‐2) in the lung actually expressed rather low levels of *ACE2*. It was, therefore, speculated that SARS‐CoV‐2 infection may depend on coreceptors or other auxiliary membrane proteins.^8^ For example, Hoffmann et al^6^ demonstrated that SARS‐CoV‐2 uses ACE2 for entry and requires the cellular protease TMPRSS2 for priming. Furthermore, potential candidate coreceptors were suggested to facilitate virus entry including glutamyl aminopeptidase (*ENPEP*), alanyl aminopeptidase (*ANPEP*), and dipeptidyl peptidase 4 (*DPP4*),^8^ the latter two being established coreceptors for the human coronavirus (hCov) 229E^10^ and hCov‐EMC.^11^ Currently, it is not clear whether conjunctival epithelia express *ACE2* or potential auxiliary proteins and coreceptors such as *TMPRSS2, ANPEP, DPP4*, and *ENPEP*.

In view of the lacking data, this study aims to explore the expression levels of *ACE2* and its potential coreceptors such as *ANPEP, DPP4, ENPEP*, and *TMPRSS2* in transcriptome data of conjunctival samples. We show that *ACE2* and its potential coreceptors are not significantly expressed in the human conjunctiva, which suggests a very low probability of SARS‐CoV‐2 propagation in the conjunctiva.

## 2 METHODS

### 2.1 Patients

To obtain information on the transcription of *ACE2* and associated molecules required for cell entry by SARS‐CoV‐2, existing datasets of 38 conjunctival samples from 38 patients were included in this study. The samples comprised twelve healthy conjunctival tissue specimens from twelve subjects who underwent buckle or 20‐gauge vitrectomy surgery for retinal detachment as well as twelve conjunctival melanoma, seven conjunctival squamous cell carcinoma and seven conjunctival papilloma specimens that had been treated at the Eye Centre of the University Freiburg from 1996 to 2017. Another eight healthy conjunctival samples from eight subjects undergoing retinal detachment surgery were included for immunohistochemical staining. All specimens contained conjunctival epithelium and subconjunctival connective tissue. All tissue samples were analyzed in an anonymized manner. Institutional Review Board (IRB)/Ethics Committee approval had been obtained for specimen acquisition, use, and data generation.

### 2.2 Tissue processing, library preparation, and sequencing

Formalin fixation and paraffin embedding of ocular samples were performed immediately after tissue excision according to routine protocols, as previously described^12^, ^13^ Following routine histological staining, each specimen's histological diagnosis was made by two experienced ophthalmic pathologists. Fifteen 4‐µm‐thick FFPE conjunctival sections were collected and stored in tubes before RNA extraction. RNA isolation from FFPE specimens was carried out as previously described.^12^ Briefly, total RNA was extracted from FFPE samples using the Quick‐RNA FFPE Kit (Zymo Research, Irvine, California). Following DNAse I digestion using the Baseline‐ZERO Kit (Epicentre, Madison, WI), the RNA concentration was quantified using the Qubit RNA HS Assay Kit on a Qubit Fluorometer (Life Technologies, Carlsbad, CA). RNA quality was determined via the RNA Pico Sensitivity Assay on a LabChip GXII Touch (PerkinElmer, Waltham, MA). RNA sequencing was performed using a massive analysis of complementary DNA ends (MACE), a 3′ RNA sequencing method, as previously described.^12^ The barcoded libraries comprising unique molecule identifiers were sequenced on the NextSeq 500 (Illumina) with 1× 75 bp. PCR bias was removed using unique molecular identifiers.

### 2.3 Data analysis

Sequencing data were uploaded to and analyzed on the Galaxy web platform (usegalaxy.eu)^14^ as previously described.^15^ Quality control was achieved with FastQC Galaxy Version 0.72 (http://www.bioinformatics.babraham.ac.uk/projects/fastqc/ last access on 11/19/2019). Reads were mapped to the human reference genome (hg38, Gencode 32, https://www.gencodegenes.org/human/releases.html) with RNA STAR Galaxy Version 2.6.0b‐2^16^ (default parameters) using the Gencode annotation file (Gencode 32). Reads mapped to the human reference genome were quantified using featureCounts Galaxy Version 1.6.4^17^ (default parameters). The output of featureCounts was imported to RStudio (Version 1.2.1335, R Version 3.5.3). Transcripts per million were calculated based on the output of featureCounts (assigned reads and feature‐length), as previously described.^18^ Gene symbols and gene types were identified based on ENSEMBL release 98 (Human genes, GRCh38.p12, download on 11/19/2019).^19^ Transcripts per million for *ACE2, CD81, LDLR, ANPEP, DPP4, ENPEP, TMPRSS2, KRT19*, and *KRT13* were extracted from the data and plotted as boxplots using ggplot2.^20^

### 2.4 Immunohistochemistry

ACE2 immunohistochemistry was performed as previously described^12^, ^21^. In brief, slides were exposed to citrate buffer at 95°C in a steamer for 30 minutes to achieve antigen recovery. Following incubation with blocking solution (Ultravision Block; Thermo Fisher Scientific), the sections were incubated with two different primary monoclonal mouse antibodies against human ACE2 (AMAB91262; clone CL4035; Sigma and MAB933; clone 171606; R&D Systems) diluted in a 1:2000 proportion for AMAB91262 for 1 hour at room temperature and 1:50 for MAB933 for 24 hours at 4°C. After extensive washing, a secondary polyclonal antibody labeled with biotin (#5570‐0006; SeraCare) was added for 30 minutes at room temperature. Finally, streptavidin‐alkaline phosphatase (#71‐00‐45; KPL) was added for 30 minutes. Vector Red AP Substrate Kit I (#SK‐5100; Vector) was used as a chromogen and applied to the sections for 7 minutes. Nuclear counterstaining was performed using hematoxylin (Mayers LOT: 18433). Sections of the human kidney were used as positive controls. Tissue samples undergoing the same procedure but without primary antibodies served as negative controls. All sections were imaged using a Hamamatsu NanoZoomer S60 (Hamamatsu Photonics, Herrsching, Germany).

## 3 RESULTS

### 3.1 Patient characteristics

In total, the transcriptome of 38 samples from 38 patients was analyzed in this study. Furthermore, eight healthy conjunctival samples from eight patients were included for immunohistochemistry. A detailed summary of the patient's characteristics is summarized in Table 1.

**Table 1:** Patient characteristics

	RNA sequencing analysis				IHC
Group	Healthy conjunctiva	Conjunctival melanoma	Conjunctival SCC	Conjunctival papilloma	Healthy conjunctiva
n (M/F)	12 (10/2)	12 (3/9)	7 (5/2)	7 (3/4)	8 (6/2)
Age at surgery, y	55.9 (43‐69)	58.9 (27‐85)	69.6 (45‐80)	37.1 (5‐66)	56.0 (43‐68)

*Note*: Data are presented as mean (range) or as absolute (relative) numbers.

Abbreviations: IHC, immunohistochemistry; SCC, squamous cell carcinoma.

### 3.2 ACE2 expression in conjunctival samples

**Figure 1:**
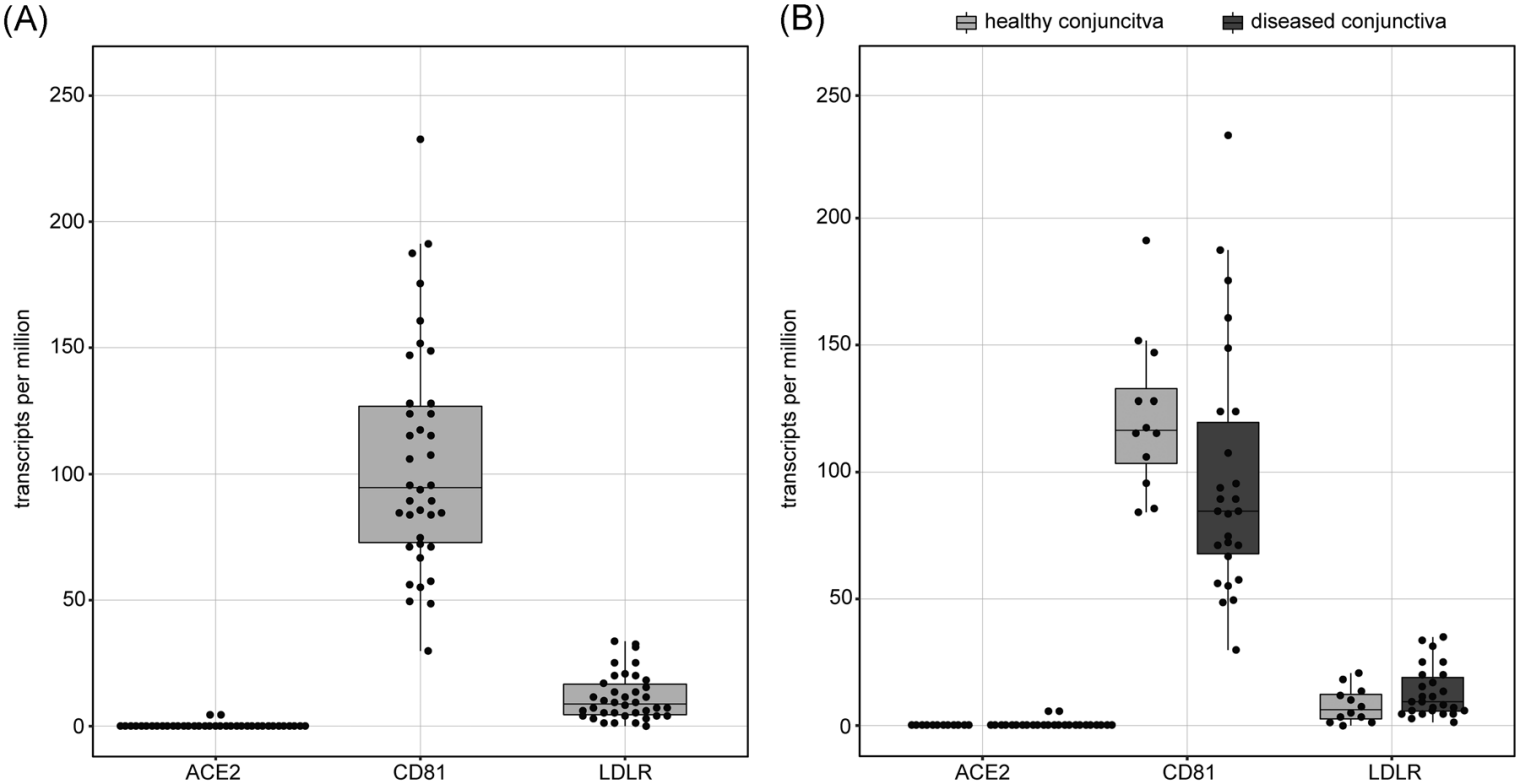
Box‐Plot showing *ACE2, CD81*, and *LDLR* expression values of all analyzed conjunctival samples (n = 38). A, Conjunctival samples do not express the SARS‐CoV‐2 receptor *ACE2* but *CD81* and *LDLR* which are common receptors for hepatitis C viruses. B, Subgroup analysis demonstrating that *ACE2* expression is similarly low in healthy and in diseased conjunctival samples, while *CD81* and *LDLR* expression is increased among all samples. Each dot represents one sample. ACE2, angiotensin‐converting enzyme 2; SARS‐CoV‐2, severe acute respiratory syndrome coronavirus‐2

Using MACE RNA sequencing, a mean number of 4.9 million raw reads (interquartile range [IQR]: 2.3‐6.2) was obtained in all 38 conjunctival samples. As a reference, the expression of known conjunctival epithelial markers was analyzed. The median expression for *KRT19* and *KRT13* was 4715.6 transcripts per million (TPM, IQR: 2085.9‐6309.5) and 29.6 TPM (IQR: 9.6‐82.3), indicating significant expression of these markers in our samples (Figure S1). The SARS‐CoV‐2 receptor *ACE2*, however, showed hardly any expression in conjunctival samples (median: 0.0 TPM, IQR: 0.0‐0.0, min: 0.0 TPM, max: 1.7 TPM). Of the 38 analyzed conjunctival samples, 35 samples (92.1%) revealed no *ACE2* transcripts, and three of them negligible amounts of 0.1, 1.1, and 1.7 TPM, respectively. A subgroup analysis of all conjunctival samples revealed that *ACE2* expression was not only insignificant in healthy conjunctival tissue (one sample with 0.1 TPM) but also in altered conjunctival samples such as conjunctival papilloma (one sample with 1.7 TPM), squamous cell carcinoma (all samples with 0.0 TPM) or melanoma (one sample with 1.1 TPM). In contrast to the above‐mentioned data, we found considerable expression of *CD81* and *LDLR* in conjunctival samples (*CD81*: 94.6 [72.9‐126.9], *LDLR*: 8.8 [4.5‐16.6], median TPM [IQR]; Figure 1A,B) which represent the targets of hepatitis C viruses for which a transconjunctival transfection has been reported.^21^

**Figure 2:**
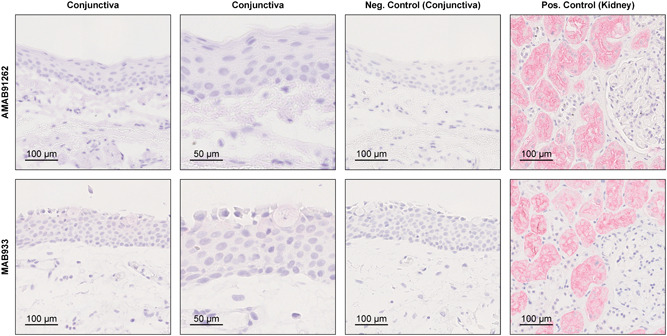
Representative immunohistochemical images of an ACE2 staining of conjunctival samples with two different monoclonal antibodies (upper row AMAB91262, lower row MAB933). While the kidney tissue shows a strong ACE2 staining, healthy conjunctival samples (n = 8) show a negligible immunoreactivity. For the negative control the primary antibody was omitted. ACE2, angiotensin‐converting enzyme 2

Immunohistochemical staining of eight healthy conjunctival samples confirmed a negligible ACE2 expression in all analyzed samples (Figure 2). In contrast to a strong staining in the kidney, none of the analyzed conjunctival samples revealed a significant ACE2 staining. Both primary antibodies used in this study, which bind different ACE2 epitopes, showed a very similar staining pattern thus reinforcing the results. Interestingly, the MAB933 antibody showed an incidental staining of a few goblet cells in four out of eight samples which were less pronounced when using the AMAB91262 antibody.

### 3.3 ANPEP, DPP4, ENPEP, and TMPRSS2 in conjunctival samples

**Figure 3:**
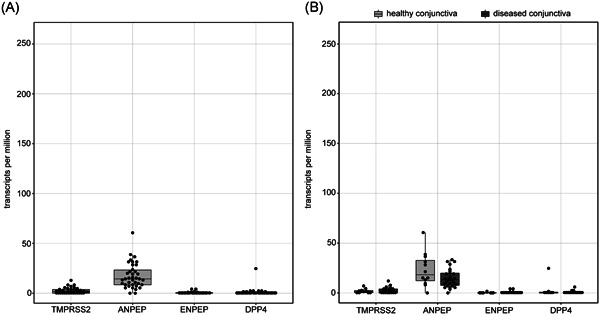
Boxplots showing *TMPRSS2, ANPEP, ENPEP*, and *DPP4* expression values of all analyzed conjunctival samples (n = 38). A, Conjunctival samples express minor quantities of ANPEP and insignificant numbers of *TMPRSS2, ENPEP*, and *DPP4* RNA. B, Subgroup analysis demonstrating that *TMPRSS2, ANPEP, ENPEP*, and *DPP4* expression is similarly low in healthy and in diseased conjunctival samples. Each dot represents one sample. ANPEP, alanyl aminopeptidase; DPP4, dipeptidyl peptidase 4; ENPEP, glutamyl aminopeptidase

Next, we assessed the transcription levels of other potential coreceptors and auxiliary proteins for SARS‐CoV‐2 infection such as *ANPEP, DPP4, ENPEP*, and *TMPRSS2*. Similar to the above mentioned results, conjunctival samples showed hardly any expression of *DPP4* (median: 0.0 TPM, IQR: 0.0‐0.8 TPM, min: 0.0 TPM, max: 24.7 TPM, samples with 0 TPM: 55.3%), *ENPEP* (median: 0.0 TPM, IQR: 0.0‐0.50 TPM, min: 0.0 TPM, max: 4.2 TPM, samples with 0 TPM: 55.3%) and *TMPRSS2* (median: 1.7 TPM, IQR: 0.0‐3.8 TPM, min: 0.0 TPM, max: 12.0 TPM, samples with 0 TPM: 28.9%; Figure 3A), whereas there was a slightly increased but still insignificant expression of *ANPEP* compared to the three factors mentioned before (median: 14.4 TPM, IQR: 8.5‐23.3 TPM, min: 0.0 TPM, max: 60.6 TPM, samples with 0 TPM: 5.3%). Subgroup analysis revealed, that expression of *DPP4, ENPEP, TMPRSS2*, and *ANPEP* was comparably low in healthy conjunctiva (*DPP4*: 0.2 [0.0‐0.8], *ENPEP*: 0.0 [0.0‐0.2], *TMPRSS2*: 1.6 [0.3‐2.3], *ANPEP*: 18.3 [12.3‐32.7], median TPM [IQR]) when compared to diseased conjunctiva (*DPP4*: 0.0 [0.0‐0.8], *ENPEP*: 0.1 [0.0‐0.6], *TMPRSS2*: 2.0 [0.0‐4.0], *ANPEP*: 13.4 [7.7‐19.9], median TPM [IQR]; Figure 3B).

## 4 DISCUSSION

The possible transmission of SARS‐CoV‐2 through the conjunctiva is controversial and has significant public health implications. Some recent reports have speculated that SARS‐CoV‐2 can be transmitted via the mucous membranes including the conjunctiva,^3^, ^4^, ^5^ and suggested that all ophthalmologists are at increased risk and should wear protective goggles when investigating suspected cases.^5^ These reports highlight the need for further research to investigate the possible transmission of SARS‐CoV‐2 via the conjunctiva, especially in the light of recent findings that 0.9% of all COVID‐19 patients exhibit signs of conjunctival congestion.^2^

This study shows that *ACE2*, which is the main receptor for SARS‐CoV‐2,^6^ is not significantly expressed in healthy and diseased human conjunctival samples. The results of this study are consistent with previously published transcriptomic datasets showing a very low expression of ACE2 in conjunctival samples.^22^ In line with the low ACE2 mRNA levels, we found a negligible ACE2 immunoreactivity, indicating insignificant ACE2 protein expression in the conjunctiva. Our results differ from a recently published study by Zhang et al^23^ which postulates a strong ACE2 immune reactivity in the conjunctiva. This discrepancy may be related to the fact that Zhang et al^23^ used a polyclonal antibody which, in contrast to monoclonal antibodies, more often causes an unspecific staining. According to the recommendations of the International Working Group for the Validation of Antibodies,^24^ which was formed with representatives of several large academic institutions, we, therefore, used two different monoclonal primary antibodies in this study. Both antibodies led to a strong immunoreactivity in the kidney but an insignificant staining in the conjunctiva, indicating an irrelevant ACE2 protein content in the healthy conjunctiva. Interestingly, when using the antibody MAB933 very few ACE2‐positive goblet cells were found in four of eight samples, which was less pronounced when using the AMAB91262 antibody. This staining may be explained by unspecific cross‐reactivity of the antibodies or may indicate an occasional expression of ACE2 in goblet cells as described for the respiratory tract of cats.^25^ Furthermore, our data demonstrate that also candidate comediators of SARS‐CoV‐2 entry such as *ENPEP, ANPEP, DPP4*, and *TMPRSS2* are not substantially transcribed in conjunctival tissues rendering the likelihood of a conjunctival entry and replication of SARS‐CoV‐2 even more unlikely. Thus, SARS‐CoV‐2 may differ significantly from other viral infections in which conjunctival infection and transmission are possible,^21^ as for example hepatitis C viruses, which can infect the conjunctiva and the organism by binding to *CD81* and *LDLR*, which according to our data are expressed in conjunctival tissue.

Our results are consistent with recent studies showing rare evidence of SARS‐CoV‐2 in conjunctival smears. Zhou et al^26^ recently analyzed 67 cases of confirmed or suspected cases of SARS‐CoV‐2 and reported that conjunctival swab samples from only one patient yielded positive PCR results and two patients yielded probable positive PCR results. None of the three patients had ocular symptoms. Similarly, Xia et al^27^ investigated a total of 30 patients with confirmed SARS‐CoV‐2 detection in sputum samples and reported that only one of them revealed SARS‐CoV‐2 RNA in the conjunctival swab and exhibited signs of conjunctivitis including conjunctival congestion and aqueous secretion. The detection of SARS‐CoV‐2 RNA in conjunctival swabs, however, does not necessarily implicate that SARS‐CoV‐2 can enter conjunctival epithelial cells and replicate in this location. It is possible that SARS‐CoV‐2 reaches the conjunctiva by aerosol or in the context of viremia during the acute phase of the disease^28^, ^29^ and that the detection of SARS‐CoV‐2 RNA in tears and conjunctival secretions of the SARS‐CoV‐2 patient complicated with conjunctivitis is a coinciding event, rather than caused by SARS‐CoV‐2 infection of the conjunctiva.^29^ Nevertheless, sufficient sample size and well‐characterized studies are required to obtain more evidence. A limitation of this study is that the approach of bulk RNA sequencing employed only reflects the average gene expression from the entire conjunctiva and cannot reveal important biological aspects of cell heterogeneity. Future single‐cell RNASeq studies are warranted to determine whether a subpopulation of conjunctival cells expresses ACE2 as reported for type II alveolar cells in the lung.^9^ However, since the majority of samples in our bulk RNA sequencing analysis revealed zero ACE2 transcripts and only a negligible immunoreactivity for ACE2 protein was detected, we consider this possibility unlikely.

Although our data indicate that *ACE2* and auxiliary factors are not transcribed in conjunctival tissue, suggesting a low likelihood of conjunctival SARS‐CoV‐2 replication, this does not diminish the requirement of protective efforts by all healthcare professionals, not just ophthalmologists, when treating patients with known or suspected SARS‐CoV‐2 infection. Inoculation of SARS‐CoV‐2 could occur by tears transporting the virus through the nasolacrimal drainage system towards the nasopharynx. In addition to an effective mask that covers the mouth and nose, protective goggles can cover the narrow gap below the top of a mask due to the uneven surface at the base of the nose, thereby reducing the likelihood of pathogen exposure.^29^ In addition, potential currently unidentified alternative SARS‐CoV‐2 receptors may be present in the conjunctiva, which could still allow SARS‐CoV‐2 infection through the conjunctiva.

## CONFLICT OF INTERESTS

The authors declare that there are no conflict of interests.

## Supporting information


**Supplement Figure 1**: Box‐Plot showing *ACE2, KRT13*, and *KRT19* expression values of all analyzed conjunctival samples (n = 38). Conjunctival samples do not significantly express the SARS‐CoV‐2 receptor *ACE2* but high amounts of the conjunctival markers *KRT13* and *KRT19*. Each dot represents one sample
