# Supplementary figures and images for: Expression of the COVID‐19 receptor ACE2 in the human conjunctiva
Source: J Med Virol. 2020 Jul 11;92(10):2081–6. doi: 10.1002/jmv.25981 (PMC7267303; doi:10.1002/jmv.25981)

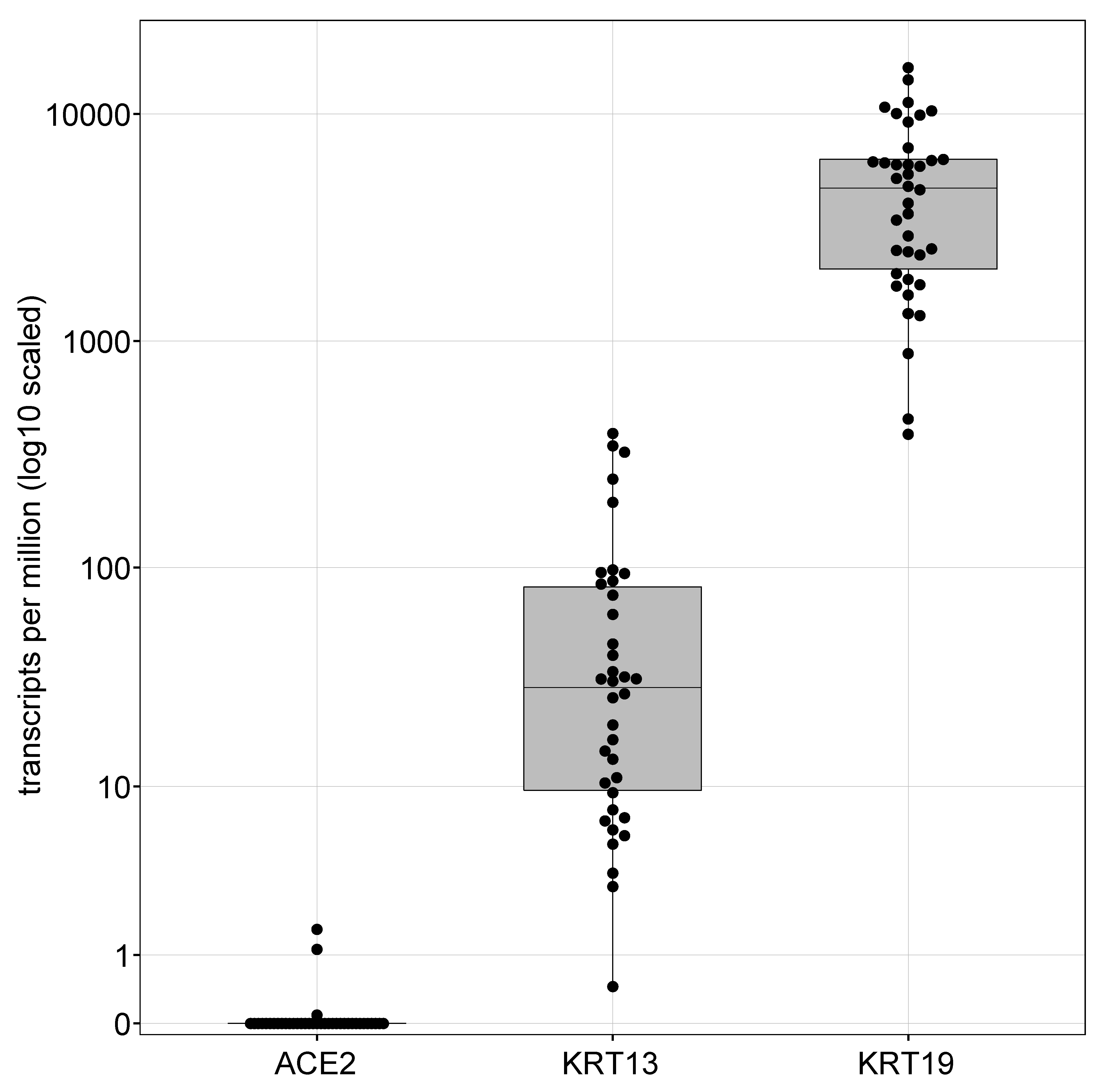

Supplement: Supplementary file 1 — Supplement Figure 1: Box‐Plot showing ACE2, KRT13, and KRT19 expression values of all analyzed conjunctival samples (n = 38). Conjunctival samples do not significantly express the SARS‐CoV‐2 receptor ACE2 but high amounts of the conjunctival markers KRT13 and KRT19. Each dot represents one sample [file JMV-92-2081-s001.tif]
